# Is serum phosphorus control related to parathyroid hormone control in dialysis patients with secondary hyperparathyroidism?

**DOI:** 10.1186/1471-2369-13-76

**Published:** 2012-08-03

**Authors:** João M Frazão, Johann Braun, Piergiorgio Messa, Bastian Dehmel, Caroline Mattin, Martin Wilkie

**Affiliations:** 1 Department of Nephrology, Hospital de S. João, Medical School & Nephrology Research & Development Unit, University of Porto, Porto, Portugal; 2 KHz Kuratorium fur Dialyse und Nieren transplantation, Nurnberg, Germany; 3 Fondazione IRCCS Ospedale Maggiore Policlinico Milano, Milan, Italy; 4 Amgen Europe GmbH, Zug, Switzerland; 5 Amgen Ltd, Cambridge, UK; 6 Sheffield Kidney Institute, Sheffield Teaching Hospitals NHS Foundation Trust, Sheffield, UK

## Abstract

**Background:**

Elevated serum phosphorus (P) levels have been linked to increased morbidity and mortality in dialysis patients with secondary hyperparathyroidism (SHPT) but may be difficult to control if parathyroid hormone (PTH) is persistently elevated. We conducted a post hoc analysis of data from an earlier interventional study (OPTIMA) to explore the relationship between PTH control and serum P.

**Methods:**

The OPTIMA study randomized dialysis patients with intact PTH (iPTH) 300–799 pg/mL to receive conventional care alone (vitamin D and/or phosphate binders [PB]; n = 184) or a cinacalcet-based regimen (n = 368). For patients randomized to conventional care, investigators were allowed flexibility in using a non-cinacalcet regimen (with no specific criteria for vitamin D analogue dosage) to attain KDOQI™ targets for iPTH, P, Ca and Ca x P. For those assigned to the cinacalcet-based regimen, dosages of cinacalcet, vitamin D sterols, and PB were optimized over the first 16 weeks of the study, using a predefined treatment algorithm. The present analysis examined achievement of serum P targets (≤4.5 and ≤5.5 mg/dL) in relation to achievement of iPTH ≤300 pg/mL during the efficacy assessment phase (EAP; weeks 17–23).

**Results:**

Patients who achieved iPTH ≤ 300 pg/mL (or a reduction of ≥30% from baseline) were more likely to achieve serum P targets than those who did not, regardless of treatment group. Of those who did achieve iPTH ≤ 300 pg/mL, 43% achieved P ≤4.5 mg/dL and 70% achieved P ≤5.5 mg/dL, versus 21% and 46% of those who did not achieve iPTH ≤ 300 pg/mL. Doses of PB tended to be higher in patients not achieving serum P targets. Patients receiving cinacalcet were more likely to achieve iPTH ≤300 pg/mL than those receiving conventional care (73% *vs* 23% of patients). Logistic regression analysis identified lower baseline P, no PB use at baseline and cinacalcet treatment to be predictors of achieving P ≤4.5 mg/dL during EAP in patients above this threshold at baseline.

**Conclusions:**

This post hoc analysis found that control of serum P in dialysis patients was better when serum PTH levels were lowered effectively, regardless of treatment received.

**Trial registration:**

Clinicaltrials.gov identifier NCT00110890

## Background

Chronic kidney disease (CKD) is accompanied by progressively impaired metabolism of calcium, phosphorus and vitamin D, eventually leading to secondary hyperparathyroidism (SHPT), a clinical syndrome of abnormal mineral and bone metabolism and extraskeletal calcifications that is associated with an increased risk of bone fractures, cardiovascular morbidity and death [1,2].

As elevated serum parathyroid hormone (PTH), phosphorus and calcium have all been linked with increased morbidity and mortality in dialysis (CKD stage 5D) patients [3-7], the National Kidney Foundation’s Kidney Disease Outcomes Quality Initiative (NKF-K/DOQI™ [KDOQI^TM^) in 2003 recommended stringent targets for intact PTH (iPTH; 150–300 pg/mL), calcium (8.4–9.5 mg/dL) and phosphorus (3.5–5.5 mg/dL) for this population of patients [8]. With increasing awareness of the key contribution of elevated serum phosphorus to vascular calcification and cardiovascular morbidity [3,4,7,9-11], more recent guidelines from the Kidney Disease: Improving Global Outcomes (KDIGO) group have highlighted the importance of tight control of serum phosphorus and calcium. KDIGO suggest that levels as close to normal as possible should be aimed for in dialysis patients and that the choice of pharmacological treatment should be influenced by these parameters [12].

It has long been known that phosphorus loading in experimental renal failure can contribute to the elevated PTH levels and parathyroid gland hyperplasia that characterize SHPT [13]. Much less appreciated is the concept that SHPT-induced dissolution of bone mineral may be a significant contributor to the development of hyperphosphataemia [14], as highlighted in a recent review [15]. A recent KDOQI commentary also noted that PTH-induced mobilization of phosphorus from bone, and potentially other tissues, may contribute to hyperphosphataemia and that this pathophysiological process would not be ameliorated by phosphate binders [16].

Vitamin D sterols and phosphate binders, together with dietary phosphate restriction, have traditionally been the cornerstone of SHPT management. With traditional vitamin D-based treatments there is a trade-off between controlling PTH on one hand and elevating calcium and phosphorus on the other hand, making it difficult to achieve simultaneous control of these parameters. Vitamin D sterols promote intestinal absorption of calcium and phosphorus and, in excess, can mobilize calcium from bone. Thus, excessive calcium loading from calcium-based phosphate binders and high doses of vitamin D sterols can promote hypercalcaemia and hyperphosphataemia, which can necessitate treatment interruptions [17]. This is reflected in the small proportion of dialysis patients who succeed in achieving and maintaining the targets recommended by KDOQI for serum PTH, phosphorus and calcium [18-20].

The calcimimetic agent cinacalcet (Mimpara®/Sensipar®, Amgen Inc., Thousand Oaks, CA, USA) has a different mechanism of PTH-lowering action from vitamin D, with opposite effects on serum calcium and phosphorus levels. By enhancing the sensitivity of the parathyroid calcium-sensing receptors to extracellular calcium, cinacalcet suppresses PTH synthesis and secretion [21]. When added to a conventional treatment regimen, cinacalcet is effective in lowering serum PTH, phosphorus and calcium levels in dialysis patients [22-26]. The phase 3 OPTIMA (Open-Label, Randomized Study Using Cinacalcet to Improve Achievement of KDOQI Targets in Patients with End-Stage Renal Disease) study showed that a cinacalcet-based regimen can allow reduction of vitamin D dosage while improving mineral metabolism compared to conventional treatment [26].

We conducted a post hoc analysis of OPTIMA data to explore the relationship between the control of PTH and that of serum phosphorus in dialysis patients with SHPT.

## Methods

### OPTIMA methodology

A detailed description of the OPTIMA study methods has been presented elsewhere [26]. Briefly, haemodialysis and peritoneal dialysis patients with iPTH 300–799 pg/mL, stratified by baseline serum calcium-phosphorus ion product (Ca x P; >55 vs ≤55 mg^2^/dL^2^) and baseline vitamin D use, were randomized to receive either a cinacalcet-based regimen or conventional care (vitamin D and/or phosphate binders) in open-label fashion. For patients randomized to conventional care, investigators were allowed flexibility in using a non-cinacalcet regimen (with no specific criteria for vitamin D analogue dosage) to attain KDOQI targets for iPTH, phosphorus, calcium and Ca x P. For those assigned to the cinacalcet-based regimen, dosages of cinacalcet, vitamin D sterols and phosphate binders were optimized over the first 16 weeks, using a predefined treatment algorithm to achieve KDOQI targets.

The starting dose of cinacalcet 30 mg once daily was increased stepwise (maximum 180 mg) if iPTH was >300 pg/mL (biointact PTH >150 pg/mL), unless precluded by hypocalcaemia (corrected serum calcium <8.0 mg/dL) or adverse events. Cinacalcet dose was to be reduced if iPTH was <150 pg/mL and vitamin D sterols were not being given, or could not be further reduced. If iPTH was <150 pg/mL and/or calcium and phosphorus exceeded KDOQI targets, vitamin D dosage was reduced by approximately 50%, in sequential steps, until a minimum dose was reached (intravenous calcitriol 0.5 μg, alfacalcidol 1 μg or paricalcitol 2 μg 3 times per week [TIW] or oral calcitriol 0.25 μg TIW or alfacalcidol 0.25 μg/day). Vitamin D dose was increased if iPTH was >300 pg/mL on the maximum cinacalcet dose, or in the event of hypocalcaemia not resolving with oral calcium supplementation.

Serum PTH, calcium and phosphorus levels were measured at 2-week intervals throughout the 16-week dose optimization phase and 7-week efficacy assessment phase (EAP). The means of these parameters during the EAP were used to evaluate efficacy. PTH levels measured by biointact assay were multiplied by 1.95 to convert to iPTH values [27]. To facilitate comparison of vitamin D doses, a relative vitamin D dose was calculated by converting dosages into a multiple of the protocol-defined minimum dose for each vitamin D analogue (see values above) [26].

For names and addresses of all ethical approval committees that granted approval for OPTIMA see Additional file 1.

### Present analysis

For the present analysis, we used a relatively low serum phosphorus cut off of 4.5 mg/dL, in line with the new recommendations from KDIGO [12], as well as the earlier KDOQI target of 5.5 mg/dL [8]. For iPTH we used a cut off of 300 pg/mL, the upper limit of the range recommended by KDOQI [8]: this also falls within the broader range suggested by KDIGO [12].

The proportion of patients with serum phosphorus ≤ 4.5 or ≤5.5 mg/dL during the EAP was compared for patients who did, versus those who did not, achieve the iPTH target of ≤ 300 pg/mL during the EAP. This was done for both treatment groups combined and additionally by treatment group (conventional care or cinacalcet).

The proportion of patients with serum phosphorus ≤4.5 or ≤5.5 mg/dL during the EAP was also compared for patients who did, versus those who did not, achieve a serum PTH reduction ≥30% during the EAP, for both treatment groups combined and additionally by treatment group.

The following were also evaluated:

· Serum phosphorus, corrected calcium and alkaline phosphatase activity, at baseline and EAP, and changes from baseline to EAP, according to PTH target achievement category at EAP.

· Usage of cinacalcet, vitamin D, calcium- and aluminium-based phosphate binders and sevelamer, at baseline and end of EAP, within each PTH and serum phosphorus target category defined above.

Analysis was performed on the full analysis set (all randomized patients). A last value carried forward imputation (LVCF) method was used for patients who did not have iPTH, phosphorus or calcium values measured during the EAP. Patients who still had missing PTH or phosphorus values during the EAP after imputation (i.e. no post-baseline value) and patients with missing serum phosphorus values at baseline (N = 13)were excluded from this analysis. Key analyses were repeated based on observed data (without imputation). If vitamin D dose was missing at the end of the EAP due to early study discontinuation, the vitamin D dose at the time of discontinuation was used. Likewise, if alkaline phosphatase was missing at the end of the EAP, the last post-baseline value was carried forward.

Pearson's chi-squared test was used to compare achievement of serum phosphorus targets by PTH target achievement category (iPTH ≤ versus >300 pg/mL) and magnitude of PTH reduction (PTH reduction ≥30% versus decrease <30% or increase during the EAP). The paired *t*-test was used to assess changes in serum phosphorus and calcium and vitamin D doses from baseline to the EAP.

Logistic regression analysis was performed to explore variables associated with achievement of serum phosphorus ≤4.5 mg/dL. Variables considered were serum phosphorus; iPTH; alkaline phosphatase; use of phosphate binders, and vitamin D at baseline, age, country and randomized treatment. Variables that were found to be significant (P < 0.05) in univariate analysis were included in a multivariate analysis, using a stepwise iterative procedure.

## Results

### Patients

Results of OPTIMA are published elsewhere [26]. 184 patients were randomized to conventional care alone and 368 to cinacalcet: demographic characteristics and baseline laboratory values were similar between the treatment groups. A total of 82% of patients in the conventional care group and 76% of those randomized to cinacalcet completed the EAP. Reasons for early discontinuation included adverse events (1% conventional care *vs* 7% cinacalcet), withdrawal of consent (2% *vs* 4%), death (3% *vs* 3%), renal transplantation (3% *vs* 2%) and other causes (7% *vs* 5%) [26]. Cinacalcet was more effective than conventional care in controlling PTH: mean (SD) iPTH levels decreased from 505 (147) pg/mL at baseline to 264 (168) pg/mL during the EAP (mean change −46%) in the cinacalcet arm versus little change in the conventional care group (from 507 [143] to 519 [281] pg/mL; mean change +2%). Similarly, mean (SD) serum phosphorus decreased from 5.5 (1.7) mg/dL at baseline to 5.1 (1.6) mg/dL during the EAP in the cinacalcet group, but was 5.4 (1.5) mg/dL at both time points in the conventional care group [26].

Thirteen OPTIMA patients were excluded from the present analysis on the basis of missing phosphorus and/or PTH values, leaving a total of 539 patients (n = 357 cinacalcet; n = 182 conventional care). Patients showed biochemical evidence of high bone turnover, as indicated by a mean (SD) iPTH level of 505 (142) [median 487] pg/mL and alkaline phosphatase activity of 127 (92) [median 97] U/L at baseline.

### Relationship of PTH control to serum phosphorus control

Patients who achieved iPTH ≤ 300 pg/mL at the EAP were more likely to achieve either serum phosphorus ≤ 4.5 or ≤5.5 mg/dL at the EAP than those who did not (P < 0.001 for both phosphorus cut offs) [Table 1]. Among those who achieved the PTH target, the proportion of patients with serum phosphorus ≤ 4.5 mg/dL increased from 29% at baseline to 43% at EAP. However, among those who did not achieve the PTH target, the proportion of patients with serum phosphorus ≤ 4.5 mg/dL remained low (24% at baseline and 21% at EAP).A similar pattern was seen with the serum phosphorus ≤5.5 mg/dL cut off: the proportion of patients within this target increased from 57% to 70% between baseline and EAP among those who achieved the PTH target but showed little change from baseline to EAP in those who did not achieve PTH target (52 *vs 46*%) [Table 1]. Results were similar when using observed data, without imputation of missing values during the EAP.

**Table 1:** Serum phosphorus (P) category and mean serum P at baseline and after treatment (efficacy assessment phase; EAP) by iPTH achievement category during EAP

	**iPTH ≤300 pg/mL at EAP (N = 300)**	**iPTH > 300 pg/mL at EAP (N = 239)**
*Serum P category, no of patients (%)*		
≤4.5 mg/dL at baseline	88 (29)	58 (24)
≤4.5 mg/dL at EAP	130 (43)*	50 (21)
≤5.5 mg/dL at baseline	172 (57)	125 (52)
≤5.5 mg/dL at EAP	209 (70)*	111 (46)
*Serum P mean (SE) absolute values (mg/dL)*		
Baseline	5.5 (0.10)	5.5 (0.10)
EAP	4.9 (0.09)	5.6 (0.10)
Change from baseline to EAP	−0.6 (0.08)	0.1 (0.09)
p-value**	<0.001	0.19

*P < 0.001 vs subgroup with iPTH > 300 pg/mL at EAP, Pearson chi-squared test. Last value carried forward imputation (LVCF) was used for patients who did not have iPTH or phosphorus values measured during the EAP.

**Paired *t*-test.

SE: standard error.

**Figure 1:**
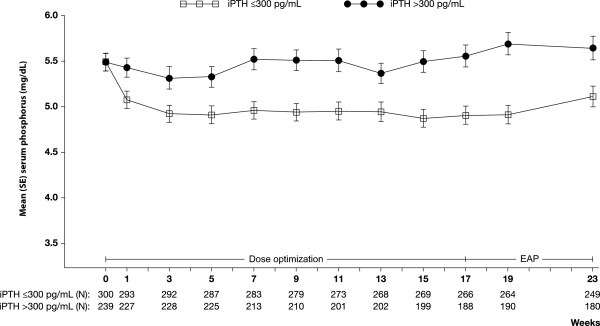
**Evolution of serum phosphorus (mean ± standard error; SE) by iPTH achievement category (≤ vs > 300 pg/mL) during the efficacy assessment phase (EAP).** Values shown on graph do not exactly match values reported in the text because imputation is used for calculating values in the text.

Patients who failed to achieve the PTH target had little change in serum phosphorus (p = 0.19), whereas those who achieved the PTH target had a significant decrease (P < 0.001). Baseline serum phosphorus levels were similar in these two subgroups of patients [Table 1; Figure 1]. Figure 1 shows that the decrease in mean serum phosphorus in those who achieved the PTH target occurred shortly after starting treatment and was sustained throughout the study.

All patients, except for two who were enrolled in error, had iPTH > 300 pg/mL at baseline: 27% and 55% of patients had serum phosphorus ≤4.5 and ≤5.5 mg/dL, respectively. Baseline iPTH was slightly higher in patients with baseline serum phosphorus > 4.5 mg/dL versus those with serum phosphorus ≤ 4.5 mg/dL (mean [SD] 513 [141] *vs* 482 [144] pg/mL; median 501 vs 457 pg/mL). A similar pattern was seen for the 5.5 mg/dL cut off (baseline mean [SD] 524 [140] *vs* 490 [143] pg/mL; median 507 vs 465 pg/mL).

**Figure 2:**
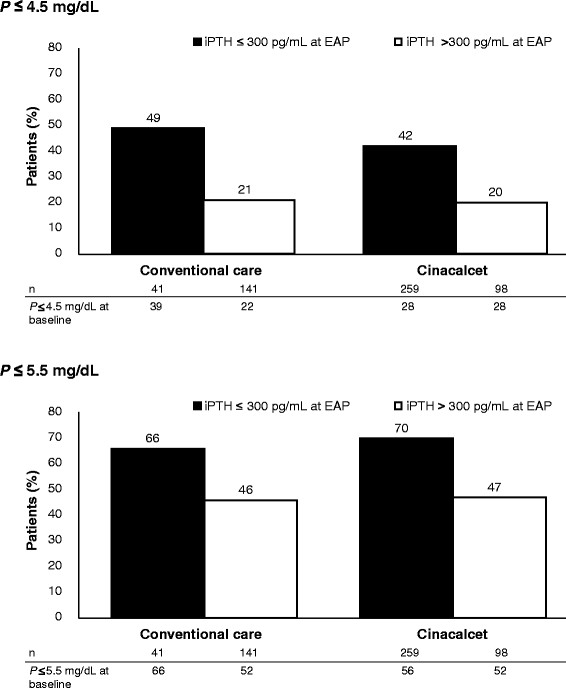
**Proportion of patients with serum phosphorus (P) (a) ≤4.5 and (b) ≤5.5 mg/dL by iPTH achievement category after treatment with cinacalcet or conventional care.** Figures show % of patients with P control (≤4.5 or ≤5.5 mg/dL) *within each PTH achievement category* during the efficacy assessment phase (EAP).

A total of 41 patients (23%) who received conventional care and 259 (73%) patients who received cinacalcet achieved iPTH ≤300 pg/mL (data not shown). Figure 2 shows the proportion of patients achieving serum phosphorus targets by achievement of PTH endpoints, separated by treatment group. Within each treatment group, achievement of serum phosphorus targets was more common in those achieving the PTH target.

Achievement of serum phosphorus targets was also more common in those who achieved a serum PTH reduction ≥30% at EAP (P < 0.001), as shown in Table 2. Results were similar when using observed data, without imputation of missing values during the EAP.

**Table 2:** Serum phosphorus (P) category at baseline and after treatment (efficacy assessment phase; EAP) by magnitude of PTH reduction at EAP

	**PTH reduction ≥30% at EAP (N = 326)**	**PTH reduction <30% or increase at EAP (N = 213)**
P ≤4.5 mg/dL at baseline, n (%)	90 (28)	56 (26)
P ≤4.5 mg/dL at EAP	131 (40)*	49 (23)
P ≤5.5 mg/dL at baseline	182 (56)	115 (54)
P ≤5.5 mg/dL at EAP	222 (68)*	98 (46)

*P < 0.001 vs subgroup with PTH reduction <30% or increase at EAP, Pearson chi-squared test. Last value carried forward imputation (LVCF) was used for patients who did not have iPTH or phosphorus values measured during the EAP.

Multivariate logistic regression analysis showed that variables associated with the probability of achieving serum phosphorus ≤4.5 mg/dL during EAP were baseline serum phosphorus, baseline iPTH, baseline phosphate binder use and randomized treatment. Patients with lower baseline serum phosphorus and iPTH, those not taking phosphate binders at baseline and those randomized to cinacalcet were more likely to achieve serum phosphorus ≤4.5 mg/dL during the EAP. When analysis was restricted to patients with serum phosphorus > 4.5 mg/dL at baseline, variables associated with the probability of achieving serum phosphorus ≤4.5 mg/dL during EAP were baseline serum phosphorus, baseline phosphate binder use and randomized treatment. Results were similar when analysis was based on observed serum phosphorus values during the EAP without imputation, although phosphate binder use was no longer significant.

### Serum calcium and alkaline phosphatase

Serum corrected calcium levels were higher in patients who did not achieve the PTH target, both at baseline and EAP. These showed a small but statistically significant decrease (P < 0.001) in both PTH target achievement categories (Table 3). In patients who received conventional care, mean serum calcium was slightly increased (+0.3 mg/dL) in those with iPTH ≤ 300 pg/mL and unchanged in those with iPTH > 300 pg/mL, at EAP. However, in those who received cinacalcet, mean serum calcium was decreased at EAP in both iPTH achievement categories (−0.7 and −0.8 mg/dL, respectively; data not shown).

**Table 3:** **Mean** (**SE) corrected serum calcium at baseline and after treatment (efficacy assessment phase; EAP) by iPTH target achievement during EAP**

**Calcium, mg/dL**	**iPTH ≤300 pg/mL at EAP (N = 300)**	**iPTH > 300 pg/mL at EAP (N = 239)**
Baseline	9.6 (0.04) [298]	9.8 (0.05) [236]
EAP	9.1 (0.05) [300]	9.5 (0.05) [239]
Change from baseline to EAP	−0.5 (0.05) [298]	−0.3 (0.05) [236]
p-value*	<0.001	<0.001

*Paired *t*-test. Last value carried forward imputation (LVCF) was used for patients who did not have iPTH or calcium values measured during the EAP.

SE: standard error.

Mean (SE) alkaline phosphatase activity at baseline was 125 (5.4) U/L (n = 242) in patients who met the PTH target and 132 (8.8) U/L (n = 144) in patients who did not meet target. Comparison of baseline and EAP values showed a decrease in both subgroups: mean (SE) -15 (3.4) U/L with PTH ≤300 pg/mL (n = 215) and −11 (5.8) U/L with PTH >300 pg/mL (n = 125) at EAP.

### Cinacalcet, vitamin D and phosphate binders

As reported previously [26], approximately two-thirds of patients in each treatment group were receiving vitamin D sterols at baseline and approximately 90% were receiving phosphate binders. Use of vitamin D increased in the conventional care group (from 66% of patients at baseline to 81% of patients at the end of the study), but showed little change in the cinacalcet group (from 68% to 73% of patients). Among patients receiving vitamin D at baseline, the mean relative dose decreased by 22% in cinacalcet-treated patients from baseline to week 23, but was virtually unchanged (3% increase) in those receiving conventional care. Cinacalcet-treated patients also required less sevelamer and aluminum-based binders but more calcium-based binders [26].

Table 4 shows mean doses of cinacalcet, vitamin D (relative doses) and phosphate binders at baseline and EAP by iPTH and serum phosphorus target category. Within both PTH strata, doses of cinacalcet and phosphate binders tended to be higher in patients who were failing to meet serum phosphorus targets, compared with those who did achieve these targets [Table 4].

**Table 4:** Usage and dose of cinacalcet, phosphate binders (mg/day) and vitamin D (relative doses)* by PTH and P subgroup at baseline and the end of the efficacy assessment phase (EAP)

**a. Target P ≤4.5 mg/dL**				
**Mean (SE) dose [% of patients]**	iPTH ≤300 pg/mL**		**iPTH > 300 pg/mL**	
	**P ≤4.5 mg/dL**	**P > 4.5 mg/dL**	**P ≤4.5 mg/dL**	**P >4.5 mg/dL**
*BASELINE*	-	-	N = 146	N = 393
Ca-based binders	-	-	1989 *(*215) [41]	1883 *(*121) [47]
Sevelamer	-	-	4137 *(*297) [48]	4876 *(*156) [55]
Vitamin D	-	-	1.5 *(*0.1) [70]	1.6 *(*0.1) [67]
Al-based binders	-	-	1874 *(*222) [18]	2450 *(*188) [20]
*EAP*	N = 130	N = 170	N = 50	N = 189
Cinacalcet	44 *(*4) [79]	54 *(*4) [84]	81 *(*15) [33]	96 *(*8) [34]
Ca-based binders	1844 *(*197) [63]	2331 *(*202) [71]	1888 *(*234) [36]	2218 *(*267) [48]
Sevelamer	4192 *(*428) [33]	4973 *(*250) [53]	4345 *(*480) [50]	4830 *(*256) [63]
Vitamin D	1.3 *(*0.1) [70]	1.6 *(*0.2) [77]	2.0 *(*0.3) [83]	1.8 *(*0.1) [78]
Al-based binders	2876 *(*605) [8]	2335 *(*319) [16]	2469 *(*557) [19]	2837 *(*369) [18]
**b. Target P ≤5.5 mg/dL**				
** *Mean(SE) dose [% of patients]* **	iPTH ≤300 pg/mL**		**iPTH > 300 pg/mL**	
	P ≤5.5 mg/dL	P >5.5 mg/dL	P ≤5.5 mg/dL	P >5.5 mg/dL
*BASELINE*	-	-	N = 297	N = 242
Ca-based binders	-	-	1880 *(*142) [45]	1944 *(*159) [45]
Sevelamer	-	-	4434 *(*195) [48]	4960 *(*196) [58]
Vitamin D	-	-	1.6 *(*0.1) [70]	1.5 *(*0.1) [65]
Al-based binders	-	-	1995 *(*199) [14]	2518 *(*215) [25]
*EAP*	N = 209	N = 91	N = 111	N = 128
Cinacalcet	46 *(*3) [83]	60 *(*6) [81]	85 *(*10) [31]	99 *(*10) [36]
Ca-based binders	1975 *(*156) [64]	2458 *(*298) [76]	2079 *(*313) [47]	2238 *(*326) [44]
Sevelamer	4454 *(*279) [40]	5168 *(*344) [57]	4175 *(*347) [51]	5103 *(*292) [68]
Vitamin D	1.4 *(*0.1) [74]	1.6 *(*0.3) [74]	2.1 *(*0.2) [82]	1.6 *(*0.2) [77]
Al-based binders	2462 *(*410) [7]	2463 *(*384) [24]	2859 *(*519) [12]	2709 *(*390) [23]

*Relative dose: dosage is based on conversion of each dose a patient received into a multiple of the defined minimum dose for a particular sterol: IV calcitriol = 0.5 μg 3 times per week (TIW), IV alfacalcidol = 1 μg TIW, IV paricalcitol = 2 μg TIW, oral calcitriol = 0.25 μg TIW, oral alfacalcidol = 0.25 μg once daily. Patients not receiving vitamin D at a timepoint are counted as having a zero dose at that timepoint. For Ca-or Aluminium-based P binders and sevelamer, dose is based on those actually receiving these agents.

**Patients were required to have iPTH 300 –799 pg/mL at baseline but two patients with PTH <300 pg/mL were enrolled in error. For simplicity, data for these patients are not shown separately.

SE: standard error.

**Figure 3:**
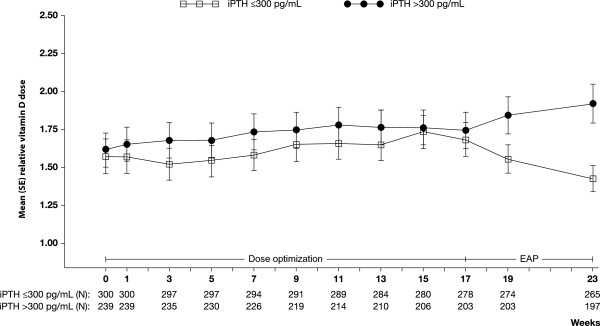
**Evolution of relative vitamin D dose (mean ± standard error; SE) by iPTH achievement category (≤ vs > 300 pg/mL) during the efficacy assessment phase (EAP).** Note that this shows vitamin D dose over time for all patients still on study. If a patient is not receiving vitamin D at a particular timepoint their relative dose will be zero and will be included as such in the calculation of mean relative dose. See text for explanation of mean relative dose. Values shown on graph do not exactly match values reported in the text because imputation is used for calculating values in the text.

The relationship of vitamin D use to serum phosphorus achievement was less consistent, but patients who were failing to meet the PTH target were receiving higher vitamin D doses [Table 4]. There was a significant increase in mean relative vitamin D dose, from 1.6 at baseline to 1.8 at the end of the EAP, in patients not achieving the iPTH target (mean change 0.2; p = 0.04). In those achieving the iPTH target, mean relative vitamin D dose decreased, from 1.6 to 1.4, although this change was not statistically significant (mean change −0.1; p = 0.18). Figure 3 shows a divergence in mean relative vitamin D dose between the two PTH target achievement categories during the EAP, although it must be noted that patient numbers decreased between baseline and EAP.

## Discussion

The present post hoc analysis of data from OPTIMA [26] showed that serum phosphorus was better controlled when PTH was lowered effectively, whether patients received cinacalcet or standard treatment. However, those receiving cinacalcet (two-thirds of patients) were more likely to achieve iPTH ≤300 pg/mL than those receiving conventional treatment (73% *vs* 23% of patients). In the subgroup of patients who achieved iPTH ≤300 pg/mL at EAP, serum phosphorus levels decreased significantly and more than 40% achieved serum phosphorus ≤ 4.5 mg/dL. The decrease in serum phosphorus occurred shortly after starting treatment and was sustained throughout the study. In contrast, the subgroup who did not achieve the PTH target did not show any improvement in serum phosphorus between baseline and EAP, either in terms of mean absolute levels or the proportion of patients within serum phosphorus targets. Only 21% had serum phosphorus ≤4.5 mg/dL at EAP.

Achievement of serum phosphorus targets was also more common in those who had a serum PTH reduction ≥30% at EAP. Logistic regression analysis identified lower baseline serum phosphorus, no phosphate binder use at baseline and cinacalcet treatment to be predictors of achieving serum phosphorus ≤4.5 mg/dL during EAP in patients who were above this threshold at baseline.

Improvement in serum phosphorus control was not related to increased phosphate binder usage overall. Indeed, usage of phosphate binders tended to be higher in patients who were failing to meet serum phosphorus targets [Table 4]. There was a significant increase in relative vitamin D dose in patients with uncontrolled PTH but this was not accompanied by a significant increase in serum phosphorus. On the other hand, a significant decrease in serum phosphorus was seen in those with controlled PTH, although a significant decrease in relative vitamin D dose was not seen in this subgroup. These findings suggest that changes in vitamin D and phosphate binders are less important than changes in PTH in this setting.

Serum corrected calcium levels were higher in patients who did not achieve the PTH target, both at baseline and EAP. It could be argued that a less suppressible PTH is accompanied by a higher serum calcium set-point and hence a higher serum calcium level. Alternatively, less suppressible PTH might have induced clinicians to use higher doses of vitamin D, with a consequent increase in serum calcium. Patients showed biochemical evidence of high bone turnover at baseline, as indicated by their elevated PTH and alkaline phosphatase values. Alkaline phosphatase activity decreased by a similar amount in the two PTH achievement categories.

As this is a post-hoc analysis, it is difficult to establish causality for the serum phosphorus changes observed in OPTIMA. There were no checks for dietary control and compliance in the study and nutritional markers were not studied. Thus, we cannot exclude the possibility that improved dietary compliance could have played a part in the serum phosphorus reduction in some patients. Bone biopsies were not performed in OPTIMA and bone turnover markers other than PTH and alkaline phosphatase were not studied. Moreover, as analyses were conducted at local laboratories, assay methods were not standardized.

PTH is known to mobilize calcium and phosphorus from bone [14] and evidence to support the hypothesis that improved PTH control leads to improved serum phosphorus control is accumulating. Substantial decreases in serum phosphorus levels have been observed in patients who undergo parathyroidectomy for advanced SHPT [28-30]. It is common to see decreases in the early postoperative period, with sustained improvement in phosphorus control thereafter. In addition, it has recently been shown that direct infusion of PTH leads to a significant rise in serum phosphorus in dialysis patients, especially in those with high-turnover bone disease [31]. This increase can be observed after several hours of infusion and during the fasting state, supporting a primary role for PTH-induced bone resorption as the mechanism for the increased phosphorus levels. Moreover, analysis of PTH evolution over time also shows that increases in PTH are associated with an increase in serum phosphorus and vice versa [32]. These findings raise the question as to whether increased serum phosphorus levels are stimulating parathyroid gland function, or whether hyperparathyroidism is leading to worsened phosphorus control via increased bone resorption. It is likely that both of these pathophysiological mechanisms operate in a vicious cycle in end-stage renal disease. Indeed, recent studies have documented the efficacy of cinacalcet in reducing increased total and bone-specific alkaline phosphatase [33].

The lower serum phosphorus cut off that we explored (≤4.5 mg/dL) is in line with new recommendations from KDIGO to aim for near-normal serum phosphorus levels in dialysis patients [12]. Although KDIGO have recommended a lowering of the phosphorus target range, they have also relaxed the PTH target range [12] for a variety of reasons, including the observation that a significant proportion of patients with iPTH in the 150–300 pg/mL range have low-turnover bone disease [34]. A recent cohort study in 22,937 dialysis patients showed that consistent control of markers of bone metabolism and disease within KDOQI targets is a strong predictor of survival in this population [19]. Compared with those who achieved target for three variables (PTH, phosphorus and calcium), the risk of death was 51%, 35–39% and 15–21% higher in patients who achieved none, one and two of these targets. For each marker, maintaining within target for one calendar quarter or less was associated with a higher mortality risk than achieving control for four quarters: 16% higher for calcium and 34% for PTH, rising to 62% higher for phosphorus [19]. Other data published since the KDOQI guidelines have shown improved outcomes in dialysis patients if PTH is controlled [35-37]. For instance, a case control study based on the US Renal Data System found a 32% reduction in hip fracture risk after parathyroidectomy [35]. Coronary artery calcification score in dialysis patients was also found to be independently associated with iPTH levels in the ADVANCE study [38]. In the CORES study in 16,173 patients, both elevated and low serum levels of calcium, phosphorus and PTH were associated with increased mortality [36].

## Conclusions

Our findings suggest that in dialysis patients with biochemical evidence of high-turnover bone disease, serum phosphorus control may be linked to PTH control: control of serum phosphorus was more difficult in patients with poorly controlled PTH. Further research is warranted to clarify these findings.

## Competing interests

J. Frazao has been a scientific consultant for, and received lecture fees from, Amgen and Genzyme. He has participated in advisory boards for Amgen, Genzyme, Abbott and Vifor. J. Braun has received honoraria from Amgen and Genzyme and participated in advisory boards for Amgen. P. Messa has given lectures supported by Amgen, Janssen-Cilag, Abbott, Novartis and Roche and participated in advisory boards for Amgen, Abbott and Novartis. M. Wilkie has given lectures supported by Amgen, Baxter and Fresenius, received honoraria from Amgen, Baxter, Fresenius and Shire and participated in advisory boards for Amgen, Baxter, Ineos and Shire. He has also participated in research funded by Amgen. B. Dehmel and C. Mattin are employees of Amgen. Amgen were involved in the design and conduct of the OPTIMA study and the present analysis and provided funding for both. Amgen staff had the opportunity to review the present manuscript.

## Authors’ contributions

This analysis was conceptualized by JF, BD and CM. JF, JB, PM and MW were investigators in the original OPTIMA study. CM provided the statistical analysis. All authors participated in the drafting and final approval of the manuscript.

## Pre-publication history

The pre-publication history for this paper can be accessed here:

http://www.biomedcentral.com/1471-2369/13/76/prepub

## Supplementary Material

Additional file 1 List of ethics committees for OPTIMA study. Click here for file
