# Supplementary material for: Is serum phosphorus control related to parathyroid hormone control in dialysis patients with secondary hyperparathyroidism?
Source: BMC Nephrol. 2012 Aug 3;13:76. doi: 10.1186/1471-2369-13-76 (PMC3473247; doi:10.1186/1471-2369-13-76)
Supplement: Additional file 1 — List of ethics committees for OPTIMA study. [file 1471-2369-13-76-S1.docx]

**LIST OF ETHICS COMMITTEES FOR OPTIMA STUDY**

**Shown as Site Number and Ethics Committee Address**

101 Ethikkommission der med. Universität Graz

Auenbruggerplatz 29

8036 Graz

Austria

102 Ethikkommission des KH der Elisabethinen Linz

Fadingerstrasse 1

4010 Linz

Austria

103 Ethikkommission des A. ö. KH der Barmherzigen Schwestern

vom heiligen Kreuz

Grieskirchnerstrasse 42

4600 Wels

Austria

104 Ethikkommission der Med. Universität Innsbruck

Innrain 43

6020 Innsbruck

Austria

106 Ethikkommission des AKH und der Universitätskliniken Wien

Borschkegasse 8b/E+F5506

1090 Wien

Austria

107 Ethikkommission der Stadt Wien

MA15, Neutorgasse 15

A-1013 Wien

Austria

132 Ethics Committee

RHMS

ATH-Baudour-Tournai

Belgium

133 Commissie voor Medische Ethiek - ZNA/O.C.M.W. Antwerpen

Lindendreef 1

2020 Antwerpen

Belgium

135 Ethics Committee

O.L. Vrouw Hospital

Aalst

Belgium

136 Ethisch Comite

Universitair Ziekenhuis

De Pintelaan 185

9000 Gent

Belgium

160 Cantonal EC Berne (KEK)

PO Box 56

3010 Berne

Switzerland

161 EC on Clinical Research

University of Lausanne

Rue du Bugnon

21-CH-1005 Lausanne

Switzerland

162 Cantonal EC Aarau

Bachstrasse 15

5001 Aarau

Switzerland

163 Ethics Committee

Dept for Internal Medicine

Building C – Floor 7

University Hospital of Geneva

Geneva

Switzerland

164 Ethics Committee (KEK)

Internal Medicine, University Hospital Zurich

Sonneggstrasse 12

8091 Zurich

Switzerland

201–236 Ethikkommission Landesärztekammer Thüringen

Postfach 100740

07707 Jena

Germany

238 Ethikkommission Landesärztekammer Thüringen

Postfach 100740

07707 Jena

Germany

300 Ethics Committee of Clinical Research

Hospital Germans Trías I Puyol

Cr del Canyet s/n

08916 Barcelona

Spain

301 Clinical Research Ethics Committee

Hospital Clínic I Provincial

C / Villarroel 170

08036 Barcelona

Spain

302 Comité Etico de Investigación Clínica

Hospital Vall d'Hebrón

Pg.Vall d'Hebrón 119-129

08035 Barcelona

Spain

304 Ethics Committee

CSU de Bellintge

Feixa Llarga s/n

08907 L'Hospitalet de Llobregat

Spain

306 Hospital Gral. De Castellón

Comité Etico de Investigación Clínica

Avenida Benicasim s/n

12004 Castellón

Spain

307 Hospital Univ. La Fe

Comité Etico de Investigación Clínica

Avda Campanar 21

46009 Valencia,

Spain

308 Comité Etico de Investigación Clínica

Hospital General Universitario de Alicante

Maestro Alonso 109

03010 Alicante

Spain

310 Comité Etico de Investigación Clínica

Hospital Univ. La Princesa

Calle Diego de León 62

28006 Madrid

Spain

312 Comité Etico de Investigación Clínica

Hospital Gregario Marãnon

28007 Madrid

Spain

315 Comité Etico de Investigación Clínica

Hospital Universitario Reina Sofía Docenia

Av. Menéndez Pidal s/n

14004 Córdoba

Spain

316 Ethics Committee

Hospital Universitario Virgen Macarena

Av. Dr Fedriani 3

41009 Sevilla

Spain

317 Ethics Committee

Hospital Regional Carlos Haya

Avda Carlos Haya s/n

29010 Málaga

Spain

318 Hospital Ntra Sra Candelaria

Comité Etico de Investigación Clínica

Ctra del Rosario 145

38010 Santa Cruz de Tenerife

Spain

319 Comité Etico de Investigación Clínica

Hospital Universitario de Canarias

Ofra s/n

38320 Laguna

Tenerife, Spain

350 Ethics Committee

A.O. Prov. Di L+F78odi Hospital Unit

Viale Savoia

26900 Lodi

Italy

351 Ethics Committee

Azienda Ospedaliera di Foggia

Viale Pinto 1

77100 Foggia

Italy

352 Ethics Committee

Azienda Ospedaliera di Foggia

Viale Pinto 1

77100 Foggia

Italy

354 Comitato Etico per la Sperimentazione dell'Azienda

Ospedaliera di Padova

Via Giustiniani 2

35128 Padova

Italy

355 Ethics Committee

Azienda Ospedaliera di Bologna

Policlinico S. Orsola Malpighi

Via Massarenti 9

40138 Bologna

Italy

356 Ethics Committee

Azienda Ospedaliera Universitaria

Viale Oxford 81

00133 Roma

Italy

357 Comititato Etico

Azienda Ospedaliera

Complesso Ospedaliero San Giovanni – Addolorata

Via dell'Amba Aradan 9

00184 Roma

Italy

359 Ethics Committee

Salvatore Maugeri Foundation

Via Maugeri 4

27100 Pavia

Italy

360 Comitato Etico Provinciale

Azienda P+F96oliclinico di Modena

Via del Pozzo 71

41100 Modena

Italy

361 Comitato Etico

Policlinico II Università di Napoli

Via Luciano Armanni 5

80138 Napoli

Italy

363 Comitato Etico

Azienda Ospedaliera

Ospedale di Circolo Fondazione

Macchi, Viale Borri 57

21100 Varese

Italy

364 Comitato Etico

Azienda Ospedaliera Mater Domini

Via T. Campanella 115

88100 Catanzaro

Italy

365 Comitato Etico

Presidio Ospedaliero Tomaselli

Via Paolo Gaifami 18

95126 Catania

Italy

366 Comitato Etico

Azienda Ospedaliera Provinciale di Lecco

Ospedale Manzoni

Via dell'Eremo 9/11

23900 Lecco

Italy

368 Comitato Etico per la Sperimentazione Clinica

Azienda Ospedaliera Careggi

Viale Pieraccini 28

50139 Firenze

Italy

370 Spettabile Comitato Etico

Azienda Ospedaliera 'G. Brotzu'

Cagliari

Italy

373 Ethics Committee

Azienda Ospedaliera 'O.C.R.' Sciacca

Via Pompei

92019 Sciacca

Italy

375 Comitato Etico

Ospedale Maggiore di Milano – IRCCS

Via F. Sforza 35

20122 Milano

Italy

400-404 STEG/METC

Saturnus 109

1115 TJ Duivendrecht

Netherlands

430-432 EC of Fresenius Medical Care

Rua Prof. Salazar de Sousa

Urbanizaçao da Quinta das Pedreiras

1750-233 Lisboa

Portugal

434-436 EC of Fresenius Medical Care

Rua Prof. Salazar de Sousa

Urbanizaçao da Quinta das Pedreiras

1750-233 Lisboa

Portugal

461-464 Northern & Yorkshire MREC

Ryhope Hospital, Ryhope

Sunderland, SR2 0LY

UK

468 Northern & Yorkshire MREC

Ryhope Hospital, Ryhope

Sunderland, SR2 0LY

UK

471 Northern & Yorkshire MREC

Ryhope Hospital, Ryhope

Sunderland, SR2 0LY

UK

472 Research Ethics Committee

The Adelaide & Meath Hospital

Tallaght

Dublin 24

Ireland

473 Northern & Yorkshire MREC

Ryhope Hospital, Ryhope

Sunderland, SR2 0LY

UK

475 Northern & Yorkshire MREC

Ryhope Hospital, Ryhope

Sunderland, SR2 0LY

UK

540 Regional Committee for Medicinal Research East-Norway

Postboks 1130

Blindern

0318 Oslo

Norway

541 Regional Committee for Medicinal Research East-Norway

Postboks 1130

Blindern

0318 Oslo

Norway

570-572 Regional Ethical Committee in Umeå

Umeå University

90187 Umeå

Sweden

600-601 Pirkanmaa Hospital District Ethics Committee

Tauh, PL2000

33521 Tampere

Finland
